# Efficiency of 2D versus 3D CT scans in maxillofacial trauma: A retrospective study

**DOI:** 10.6026/973206300210985

**Published:** 2025-05-31

**Authors:** Prasanth Panicker, Ashford Lidiya George, Rajana Mohan Kumar, Mandapathi Srujan Kumar, Feby Francis, Deepak Peter

**Affiliations:** 1 Department of Maxillofacial Facial Plastic & Reconstructive Surgery Center, Jerudong Park Medical Center, Bandar Seri Begawan, Brunei, Asia; 2 Department of Oral and Maxillofacial Surgery, Mattanur Multi Speciality Dental Clinic, Kannur, Kerala, India; 3 Department of Oral Surgery, RIPAS Hospital, Bander Seri Begawan, Brunei, Asia; 4 Department of Otorhinolaryngology, Osmania Medical College, Koti, Hyderabad - 500095, Telangana, India

**Keywords:** Maxillofacial fractures, computed tomography, 3D CT in maxillofacial trauma

## Abstract

The transition from 2D CT slices to 3D CT volume rendering reconstructions has significantly improved the precision of the trauma diagnoses and broadened the range of potential treatments. Using a 64-slice CT scanner, the fracture detection score and fracture comparative score were used to compare 2D and 3D fracture cases for detection and diagnosis. The study comprised 200 maxillofacial fracture cases. 2D CT cuts detected 100% of fractures, but 3D cuts missed 4%. In 66.66% of fracture score combinations, 2D CT slices could improve diagnosis. This study showed that 2D CT cuts are better at fracture identification and diagnosis than 3D CT reconstruction cuts. 3D CT cuts show displaced fractures overall; however, they are confined to minimally displaced fracture segments and nasal and ocular fracture locations.

## Background:

Profound facial disfigurement, be it due to trauma or any other cause, can have considerable physical and psychological repercussions on an individual. As a result, it is imperative to prioritize swift and skilful management of maxillofacial injuries, leaving no room for compromise in trauma care and aesthetic surgery [1]. Maxillofacial fractures are highly prevalent, accounting for a substantial percentage that ranges from 23% to 97% of all injuries to the facial area [2]. Facial aesthetics play a crucial role in defining an individual's identity. The increasing incidence of facial fractures, notably from automobile accidents, is a growing concern. Additional causes, including falls, sports injuries, occupational trauma and interpersonal aggression, contribute to this rise [3]. Although clinical evaluation and traditional radiography significantly aid in the diagnosis and treatment planning of maxillofacial injuries, further research may be warranted due to challenges posed by the superimposition of bony structures and difficulties in visualizing underlying fractures caused by soft tissue swelling and bleeding [4]. Computed tomography (CT) is now the preferred method in situations like maxillofacial injuries resulting from violence or road traffic accidents [5]. CT scans simplify fracture reduction, the selection and shaping of stiff reconstructive plates and surgical exploration by providing precise depictions of facial fractures and their spatial relationships. CT reduces the likelihood of non-union, malunion and other functional or aesthetic issues, minimizing the need for re-surgery caused by delays in diagnosis and treatment [6].

Surgeons face difficulty when dealing with facial injuries, given the potential for significant functional and cosmetic repercussions, typically stemming from complex origins. Because of this, precise diagnostic tools and assessment play a very critical and crucial role. For the diagnosis and screening of facial bone fractures conventional plane radiographs are essential but, chances of missing out of fracture lines or underestimating the severity of fractures like blowout, Le fort 1 and Le fort 2 are very common, also diagnostic accuracy of orbital and maxillary fractures has been reported to be 38 percentages only. Even for the most experienced surgeons and radiologists, accurate interpretation of facial fractures from conventional radiographs can be very challenging. Spiral CT overcomes these problems and drawbacks by offering fast and thin multiplanar CT in less than 20 seconds with axial, sagittal, and coronal 2-dimensional cuts and even 3-dimensional reconstructions of the same to aid fracture diagnosis [7]. The advancement from 2D CT slices to 3D CT volume rendering reconstructions has notably enhanced the accuracy of diagnosing these injuries and expanded the array of treatment options available [8]. Several studies demonstrate the use of 3D CT in the treatment of craniofacial injuries. Still, none of them examined the benefits and drawbacks of doing so in comparison to traditional 2D CT slices. Therefore, it is of interest to compare the conventional 2D-CT slices and 3D CT reconstructed images in maxillofacial fractures based on fracture detection and diagnostic accuracy.

## Materials and Methods:

This retrospective observational study encompassed CT Scans of patients between the ages of 10 - 60 visiting two tertiary health care institutions diagnosed with maxillofacial fractures between the year of 2018- 2021 for two years. This research was complied with the principles of the Declaration of Helsinki. Confidentiality of the study participants was maintained. The sample size was estimated using the formula n = 4pq/d2, where p = prevalence from a previous study [9] (43% fractures in both upper and middle third), d = margin of error of 20%, and q = 1-p. The minimum estimated sample of 128. However, the sample size was increased to 200 patients with maxillofacial fractures to compensate for any artifacts and image distortions. The inclusion criteria were that CT scan cuts of 200 patients diagnosed with maxillofacial fractures were studied and compared, who were reported to the Emergency Department and in the Oral and Maxillofacial Surgery OPD, between the age group of 10 to 60 years. The CT scans of patients with image artifacts were excluded. A simple random sampling technique was employed. The copy of CT scans, cuts (both 2D and 3D slices) of Patients reported for any maxillofacial trauma treatment was collected from the radiology department.

The method consisted of 2 steps:

[1] Confirmation of fracture detection in both the 2D and 3D cuts.

[2] Comparison of fracture presentation in both cuts. (2D and 3D) based on diagnostic information.

In each case, the conventional 2D cuts and 3D CT images will be analyzed under the headings of fracture sites. Cases will be grouped according to location/site of fractures. The upper third fractures included calvarial vault (frontal, temporal, parietal, occipital bones), orbital roof, frontal sinus, and supra-orbital rim fractures. The middle third fractures included Zygomatico-Maxillary Complex, Fronto-Zygomatic suture, Naso-Orbital Ethmoid (NOE), zygomatic arch, zygoma, nasal, maxilla, orbital floor, medial, and lateral wall fractures. The lower third mandibular fractures included Angle, ramus, condyle, body, symphysis, and parasymphysis fractures. The CT scan unit - SIEMENS was employed with 64 slices, 0.8 seconds of rotation time, 3.5 MHU tube at 32 kW power, high voltage of 80,110,130 kV, up to 400mA, z-coverage of 32*0.7mm, Maximum table load up to 307 kg. The extent of fractures and fragment displacement will be noted by the same observer. Each case will be separately studied for fracture detection from both 2D and 3D cuts using a score [0 for no fracture site detected, 1 for fracture site/sites detected], and a score will be given for both the CT cuts (2D and 3D). The positive findings and information of 2D and 3D CT cuts in each case will be analyzed, compared, and reviewed using a scoring system in Table 1 to identify which CT cut (2D or 3D) gives better information about the case for diagnosis and treatment plan.

## Statistical analysis:

All statistical procedures were performed using the Statistical Package for Social Sciences (SPSS) 20.0. Calculations for the power (80%) of the study were performed before the commencement of the study. All quantitative variables were expressed in mean and standard deviation. Qualitative variables will be expressed in percentages. The chi-square test will be used for the association between variables. The Z-test was used to determine the significant difference between 2D and 3D cut fracture detection scores. A probability value p <0.05 was considered statistically significant.

## Results:

Out of all 200 cases of maxillofacial fracture patients, a total of 608 individual fracture sites were recorded. The total number of fractures in the lower third was 138. The Total number of fractures in the middle third was 382; the total number of fractures in the upper third was 88. There were 154 male patients and 46 females. Most of the fracture cases were reported in the age groups 40-49 and 50-60, with 29% and 28% respectively. On evaluating individual fractures recorded, 63% of fractures are in the middle third region, followed by 23% in the lower third and 14% in the upper third regions. All 608 fractures were seen in 2D cuts, which accounts for 100%, but 24 fractures, which account for 4%, were not seen in 3D cuts. Out of 608 (100%) fractures, 584 (96.1%) fractures were seen in both the cuts, but 24 (4%) fractures were not identified in the 3D cuts. Those 24 (4%) fractures were only seen in 2D cuts. When comparative scores of 2D and 3D are compared frequencies of occurrence of each score are given in Table 2. No 2D cut got a score of "1" and No 3D cut got a score of "4". Out of the 18 combinations of the comparative score, four combinations showed similar diagnostic information in both 2D and 3D cuts (22.2%), two combinations showed superior diagnostic information in 3D (11.1%), a total of 12 combinations showed superior diagnostic information in 2D cuts (66.7%) (Table 3). Comparison of fracture detection score between 2D and 3D cuts displayed a p-value of 0.0001, which was <0.01, deemed to be statistically highly significant. So, the null hypothesis was rejected as there was a highly significant difference between conventional CT (2D cuts) to 3D CT reconstruction in detection, identification, and diagnostic accuracy of maxillofacial trauma. Additionally, when individual comparative scores of both 2D and 3D cuts were compared, a p-value of 0.042 (less than 0.05), which was statistically significant. That shows there was significant marked difference between the two cuts in terms of individual scoring for diagnostic information once the fracture was identified and detected in both 2D and 3D cuts (Table 4).

## Discussion:

Over the past two decades, there has been significant advancement in both radiologic diagnosis and surgical treatment of facial fractures. Previously, radiologists relied on interpreting plain films, OPG, and conventional tomography for diagnosis. While these methods provided some insight into the location and displacement of fractures, they often lacked precision due to overlapping bony structures, making accurate diagnosis challenging. The introduction of CT scans allowed for a more precise definition of the midface's bony architecture across multiple thin, two-dimensional planes, thus enhancing diagnostic accuracy. However, physicians still had to mentally reconstruct these slices into a three-dimensional understanding. Early and correct diagnosis is one of the important factors determining the success of maxillofacial fracture treatment. MDCT (Multi-Detector Computed Tomography) 2D CT is the choice when multiple fragments, displacement and degree of rotation, or any skull base fracture involvement is suspected [10] supported by the study conducted by Wang *et al.* [8] and Wahab *et al.* [11]. In this study, male predominance of facial trauma was noted, which was congruent with the study conducted by Kaur and Chopra [12]. Among 304 total fractures recorded, the middle third of the face showed the highest number, with nasal bone fractures 77.8%. The frontal bone and mandibular parasymphysis were recorded as highest in the upper third and lower third regions, respectively. In the orbit, the medial wall and the orbital floor (18.3%) were the most commonly affected sites. This is consistent with studies of orbital fractures [13]. Coronal (2D CT) reformatted images were better at assessing orbital fractures, as proved in the study conducted by Wahab *et al.* [11]. The 3D CT images were found to be inferior in the detection, extent and displacement of fractures in the orbital region when compared with 2D cuts in most patients, consistent with the study conducted by Fox *et al.* [14]. The thin bones in these regions, causing partial volume averaging resulting in "pseudoforamina" and considerable bony overlap, could explain this finding, as described in the study conducted by Kaur and Chopra [12]. Hemosinus was the most common associated finding in the patients who presented with facial trauma, which was most commonly associated with fracture of the anterior wall of the maxillary sinus. Frontal bone fractures were the next common region associated with hemosinus [13]. These were evaluated only on 2D cuts. However, Reddy *et al.* [5] determined that the identification of maxillofacial fractures, particularly displaced and comminuted types, is optimally achieved by 3D imaging. Markowitz *et al.* [15] concur with this conclusion, asserting that coronal CT is superior to axial CT for detecting mandibular fractures. Fracture patients are generally assessed by X-ray imaging; however, these two-dimensional pictures fail to accurately depict the site and displacement of intricate fractures [16, 17]. Moreover, precise placement is essential for X-ray radiography, which is frequently challenging to attain due to patient compliance, resulting in inferior quality of images [18]. Axial CT scans produce two-dimensional images that eliminate image overlap in contrast to traditional X-ray films, hence substantially enhancing detection frequencies [19, 20]. Nevertheless, they are devoid of 3D information, hindering the precise, thorough, and lucid evaluation of the degree and severity of jaw and facial fractures, hence constraining their clinical applicability for fracture diagnosis and therapy.

Multi-slice spiral CT volumetric scanning provides rapid imaging while elucidating fracture lines and spatial correlations in intricate fractures across many planes of the jaws and face. Moreover, 2D and 3D reconstructions provide a more thorough and spatially precise depiction of the images. Research indicates that 3D reconstruction techniques offer substantial benefits over 2D reconstruction in the diagnosis of maxillofacial fractures, especially in evaluating the degree of bone injury and fracture displacement. It can function as the benchmark inquiry for many maxillofacial fractures [21]. Nonetheless, 3D CT reconstruction necessitates sophisticated post-processing workstations and competent technical proficiency and image interpretation skills, which may pose difficulties for general medical practitioners in conducting detailed analyses, hence requiring dependence on specialised radiologists [22]. In this study Parasymphysis (36.2%) region of the mandible had with highest frequency among lower third fractures, for which 3D CT gave more picturisation of the segments, but the position and angulations of the displaced condyle were accurately read with 2D cuts. Undisplaced fractures are commonly missed in 3D CT cuts; in this study total of 12 fracture sites were not appreciated in 3D CT, which were viewed in 2D cuts. In addition to the hard tissue findings, 2D cuts share a picture of soft tissue associated with the fracture segments, which allows the surgeon to correlate the vital structures associated with the fracture for better diagnosis and treatment plan, especially seen with the nasal and orbital regions. Furthermore, because 3DCT offers a better understanding and picturisation of fracture comminution, surgeons can anticipate when standard internal fixation techniques might not be feasible or not, potentially necessitating primary bone grafting needed or not. This enhances technical outcomes, streamlines efficiency, and reduces operative time. Additionally, we found that 3DCT is invaluable in teaching facial fracture classification and management concepts.

## Conclusion:

2D CT cuts provide excellent spatial resolution in the detection of maxillofacial fractures. This offers a superior assessment that correlates soft tissue findings with fractures in the maxillofacial region. 3D CT images provide a much better perception of the fracture lines and the displacement of the bony fragments. This will improve communication with adequate information to the referring surgeon. Thus, this study demonstrates that 2D CT cuts stand superior to 3D CT reconstruction cuts in terms of both fracture detection as well as providing fracture diagnostic information.

## Figures and Tables

**Table 1:** Comparative scoring system: Score-3D and 2D cuts assessment

**Scoring system**	**3D**	**2D**
1	Inferior	Inferior
2	Similar	Similar
3	Superior- similar information is more rapidly assessed	Superior- similar information is more rapidly assessed
4	Superior- additional conceptual information provided	Superior- additional conceptual information provided

**Table 2:** Frequencies of comparative scores of 2D and 3D cut images

**2D cut comparative score**		**3D cut comparative score**			**Total**
		**1**	**2**	**3**	
2	Observed	2	4	372	378
	% within row	0.5	1.1	98.4	100
	% within column	2.4	2.9	96.9	62.2
3	Observed	8	104	4	116
	% within row	6.9	89.7	3.4	100
	% within column	9.5	74.3	1	19.1
4	Observed	74	32	8	114
	% within row	64.9	28.1	7	100
	% within column	88.1	22.9	2.1	18.8
Total	Observed	84	140	384	608
	% within row	13.8	23	63.2	100
	% within column	100	100	100	100

**Table 3:** CT cut superiority percentage of fracture score combinations in providing diagnostic information

**Score Combinations**	**Percentage**	**Inference**
2D- "2" (378) & 3D- "3"(372)	98.40%	3D superior
2D- "2" (378) & 3D- "2"(4)	1.10%	Both similar
2D- "2" (378) & 3D- "1"(2)	0.50%	2D superior
2D- "3" (116) & 3D- "3"(4)	3.40%	Both similar
2D- "3" (116) & 3D- "2"(104)	89.70%	2D superior
2D- "3" (116) & 3D- "1"(8)	6.90%	2D superior
2D- "4" (114) & 3D- "3"(8)	7.00%	2D superior
2D- "4" (114) & 3D- "2"(32)	28.10%	2D superior
2D- "4" (114) & 3D- "1"(74)	64.90%	2D superior
3D- "1" (84) & 2D- "2"(2)	2.40%	2D superior
3D- "1" (84) & 2D- "3"(8)	9.50%	2D superior
3D- "1" (84) & 2D- "4"(74)	88.10%	2D superior
3D- "2" (140) & 2D- "2"(4)	3%	both similar
3D- "2" (140) & 2D- "3"(104)	74%	2D superior
3D- "2" (140) & 2D- "4"(32)	23%	2D superior
3D- "3" (384) & 2D- "2"(372)	97%	3D superior
3D- "3" (384) & 2D- "3"(4)	1.00%	both similar
3D- "3" (384) & 2D- "4"(8)	2.00%	2D superior

**Table 4:** Comparison of fracture detection score between 2D & 3D cuts

	**N**	**Mean ± SD**	**Median**	**Mean difference**	**Statistic**	**df**	**p-value**
2D cut fracture detection score	608	1.04 ± 0.02	1	0.07	8.18	1214	<0.0001**
3D cut fracture detection score	608	0.97 ± 0.21	1				
2D cut comparative score	608	2.6 ± 0.81	2	0.09	2.04	1214	0.042*
3D cut comparative score	608	2.51 ± 0.73	3				
**p<0.01 - statistically highly significant;
*p<0.05 - statistically significant
